# A Case Report of Cardiac Tamponade

**DOI:** 10.21980/J8J644

**Published:** 2020-04-19

**Authors:** Derek JC Hunt, Kevin McLendon, Matthew Wiggins

**Affiliations:** * Merit Health Wesley, Department of Emergency Medicine, Hattiesburg, MS

## Abstract

Cardiac tamponade is a rare and life-threatening condition that can be caused by trauma and various medical conditions. Failure to recognize and repair it quickly could lead to significant morbidity or even death. This case demonstrates the electrocardiogram (ECG) findings of low voltage QRS and electrical alternans in cardiac tamponade. It also highlights the classic ultrasound (US) findings of pericardial effusion and right ventricular collapse during diastole in cardiac tamponade. Classic physical exam findings of cardiac tamponade include Beck's Triad (jugular venous distention, hypotension, and muffled heart sounds) and pulsus paradoxus. This patient only had jugular venous distention and pulsus paradoxus. The case is centered on a 52-year-old male who presented with shortness of breath, wheezing, and a productive cough with streaks of blood. A CT chest was performed which revealed a large pericardial effusion, right upper lobe lung mass, and bilateral pulmonary emboli. A bedside transthoracic echocardiogram was then performed which confirmed the large effusion as well as right ventricular collapse during diastole. Cardiothoracic surgery and interventional cardiology were consulted and both agreed to take the patient to the cardiac catheterization lab for percutaneous drainage of the effusion. Pericardiocentesis was performed and 1.7 liters of serosanguinous fluid was removed and a drain was left in place. He recovered well from the procedure and had an uneventful admission. After reviewing this case, learners should be able to recognize the diagnostic features and various causes of pericardial effusion and cardiac tamponade.

**Topics:**

Electrocardiography, echocardiography, cardiac tamponade.[Fig f1-jetem-6-2-v8][Fig f2-jetem-6-2-v8][Fig f3-jetem-6-2-v8]

**Figure f1-jetem-6-2-v8:**
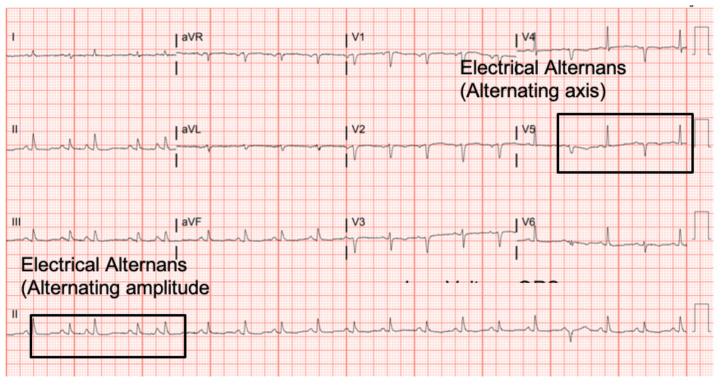


**Figure f2-jetem-6-2-v8:**
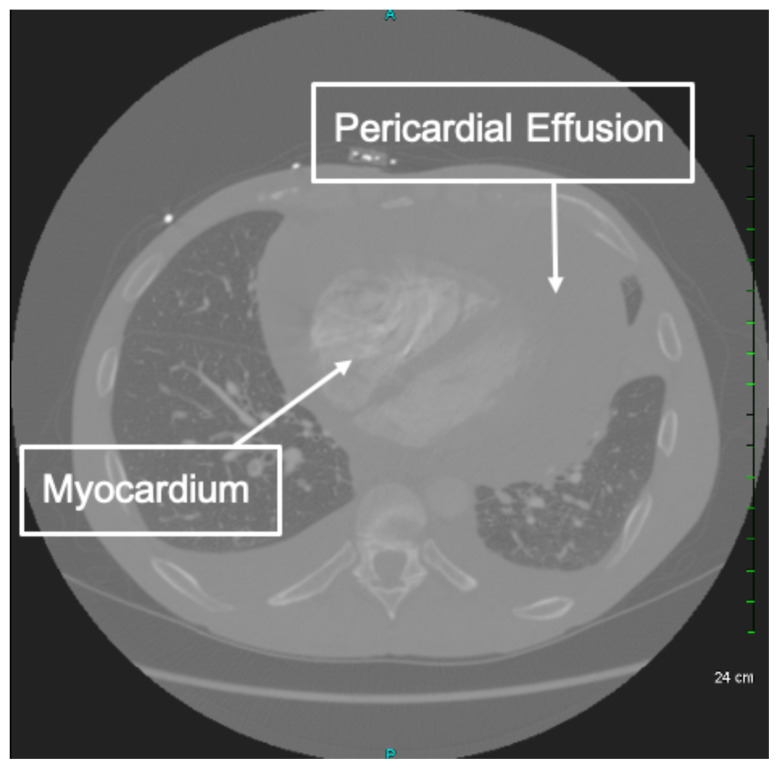


**Figure f3-jetem-6-2-v8:**
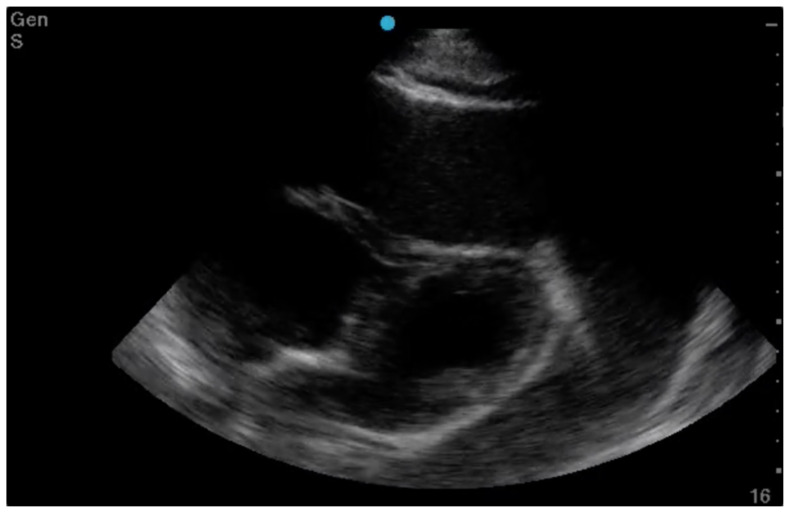
Video Link: https://youtu.be/gXLL_CdsNjA

## Brief introduction

Cardiac tamponade has various causes and occurs when excess fluid accumulates within the pericardial sac leading to compromised cardiac activity and shock. It can be caused by trauma, uremia, malignancy, infection, tuberculosis, inflammatory conditions, human immunodeficiency virus (HIV), radiation, or idiopathic. Depending on the cause, the type of fluid can be transudative, exudative, or sanguineous.^1^ The overall incidence of cardiac tamponade is unknown, but has been estimated at 2 per 10,000 per year.^2^ There are several presentations and physical exam findings associated with cardiac tamponade including tachycardia, hypotension, pericardial friction rub, jugular venous distention (JVD), and muffled heart sounds.^3^ Pulsus paradoxus, a 10mmHg decrease in systolic blood pressure during inspiration, is present during the majority of cases with cardiac tamponade. Pulsus paradoxus greater than 12mmHg has a sensitivity of 98% and specificity of 83%.^4^

## Presenting concerns and clinical findings

This is a 52-year-old male with no known medical or surgical history presenting with shortness of breath, wheezing, and a cough with blood-tinged sputum for 1 week. He smokes a half pack of cigarettes per day. He denies chest pain, fever, or chills. He does not use supplemental oxygen at home and has not had similar symptoms in the past. No reported sick exposures and he denied weight loss. He works as a painter and was sent to the emergency department from work due to shortness of breath. On initial presentation, his vital signs were temperature 97.0°F, heart rate 118, blood pressure 125/100, and oxygen saturation of 94% on room air.

## Significant findings

The patient was in noticeable respiratory distress and had oxygen saturation of 94% on room air. Bilateral jugular venous distention with severe right supraclavicular lymphadenopathy and diffuse bilateral wheezing was present. Although muffled heart sounds and hypotension are part of Beck's Triad, these were not present in this case. Electrocardiogram obtained on arrival showed a sinus tachycardia with low-voltage QRS complexes and electrical alternans. Low voltage QRS can be seen on the ECG provided and is demonstrated by the low amplitude of the QRS complexes seen on all the leads. Electrical alternans may have an alternating axis or amplitudes of the QRS complex. Alternating axis is best visualized in V4–V6 on this ECG while alternating amplitudes are seen throughout the rest of the ECG. Computed tomography angiogram (CTA) of the chest revealed a large pericardial effusion with bilateral pulmonary emboli and a right upper lobe mass. A bedside transthoracic echocardiogram (TTE) was then performed and confirmed the large effusion, but also showed right ventricular collapse during diastole, indicative of cardiac tamponade.

## Patient course

Given the patient's presentation, we were initially concerned for chronic obstructive pulmonary disease and pulmonary malignancy. He reported improvement in his symptoms after receiving nebulized albuterol/ipratropium and methylprednisolone. Once the CTA of the chest was completed, we performed the TTE to confirm our suspicion of cardiac tamponade. Since the patient's blood pressure remained stable, interventional cardiology and cardiothoracic surgery were consulted. Both agreed to take the patient to the cardiac catheterization lab for a percutaneous drainage of the effusion. During the procedure, he was noted to have pulsus paradoxus measured at 42mmHg and had a total of 1.7 liters of serosanguinous fluid removed from the pericardial sac. Cytology of the effusion showed malignant cells likely related to the right upper lobe lung mass.

## Discussion

This case demonstrates the various exam findings as well as electrocardiographic and echocardiographic findings of cardiac tamponade. Patients with cancer make up the largest group of cases with pericardial effusions that lead to tamponade. Other causes include trauma, acute infections, radiation exposure, tuberculosis, renal failure, autoimmune disease, hypothyroidism, ovarian hyperstimulation syndrome, and idiopathic.^5^ The classically taught Beck's Triad of hypotension, JVD, and muffled heart sounds was not completely present in this patient. In a study performed by Stolz et al, which featured 150 patients, none of the patients with pericardial effusions or cardiac tamponade had all three findings of Beck's Triad. Nevertheless, they did determine the sensitivity of one finding of Beck's Triad in order to diagnose pericardial tamponade to be 50%.^6^ The classically taught ECG findings of cardiac tamponade are low voltage QRS and electrical alternans. Low voltage QRS is thought to exist due to the presence of fluid that exists between the myocardium and ECG lead. Electrical alternans is present due to the swaying heart phenomenon as it sways from side-to-side within the pericardial effusion. In a recent study by Chandra et al, these ECG findings were found to be neither sensitive nor specific. Low voltage QRS was found in 29% of patients while electrical alternans was found in 23%.^7^ Similar to a lot of conditions, the ECG is a great screening tool, but cannot always be relied upon for diagnostic accuracy. In a blinded review of 155 ECGs, one study found that low voltage QRS and PR depression were suggestive of pericardial effusion and cardiac tamponade while electrical alternans was not associated with either condition.^8^ The echocardiogram can be used to confirm the diagnosis of cardiac tamponade and should be used in conjunction with a physical exam and ECG findings. The earliest US finding in tamponade is right atrial collapse during systole. An enlarged inferior vena cava has high sensitivity while diastolic collapse of the right ventricle has high specificity for cardiac tamponade.^9^ Finally, management of cardiac tamponade in the emergency department is usual resuscitation with or without pericardiocentesis. If a patient is hemodynamically stable, the procedure is best performed in a controlled environment by interventional cardiology and/or cardiothoracic surgery. This procedure can be performed with or without US, and should only be done in the ED if the patient is hemodynamically unstable. To perform the procedure, a needle should be inserted at a 45° angle just left of the xiphoid process and aimed towards the left shoulder.^5^ An ECG lead can be attached to the needle. Ventricular ectopy or ST-Elevations indicate the needle contacting the right ventricle. Complications of this procedure include myocardial perforation, bleeding, pneumothorax, and arrhythmia.

In summary, this case highlights the classically associated physical exam, ECG, and US findings in cardiac tamponade. Pulsus paradoxus, though underutilized, is a highly sensitive finding and present in up to 98% of cardiac tamponade cases. Although not diagnostic, low voltage QRS and electrical alternans may be present on ECG in cardiac tamponade, but they are poorly sensitive. Right ventricle collapse during diastole is the most specific finding of cardiac tamponade in echocardiography. Overall, the diagnosis of cardiac tamponade can be complex and should be determined by combining physical exam, ECG, and US findings.

## Supplementary Information












