# Supplementary figures and images for: A Case Report of Cardiac Tamponade
Source: J Educ Teach Emerg Med. 2020 Apr 19;6(2):V8–V12. doi: 10.21980/J8J644 (PMC10332784; doi:10.21980/J8J644)

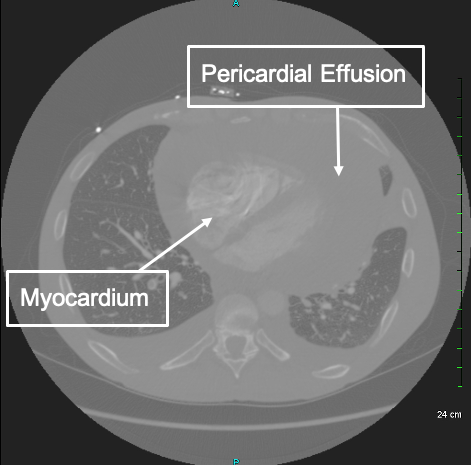

Supplement: Supplementary file 1 [file jetem-6-2-v8-supp1.png]

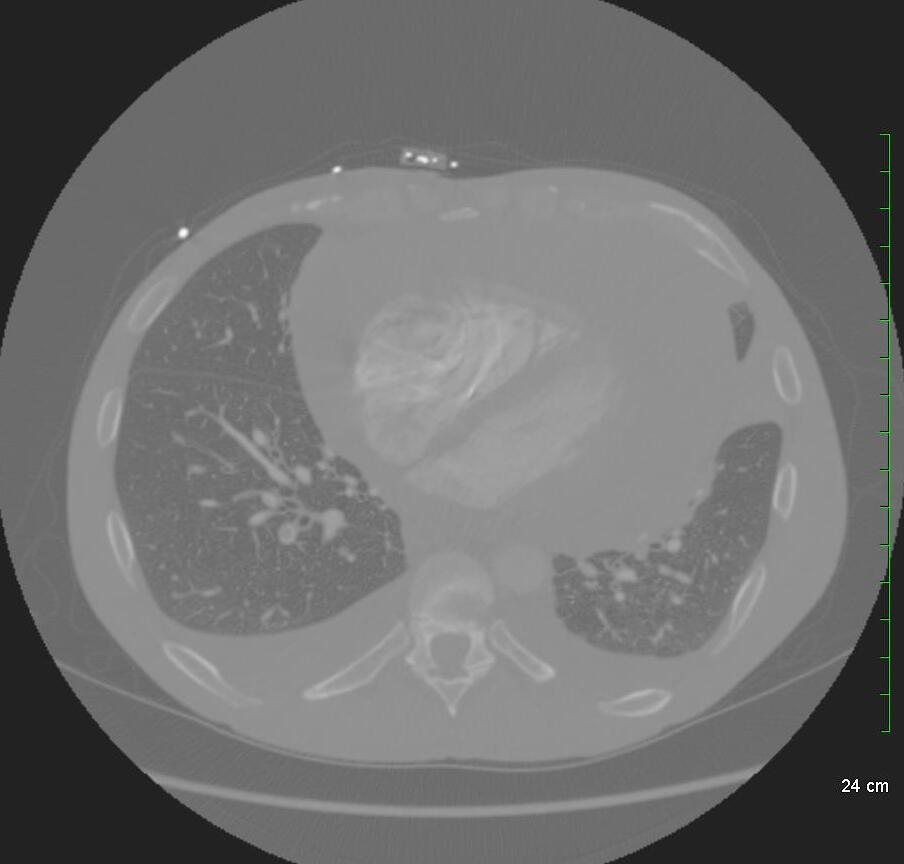

Supplement: Supplementary file 2 [file jetem-6-2-v8-supp2.jpg]

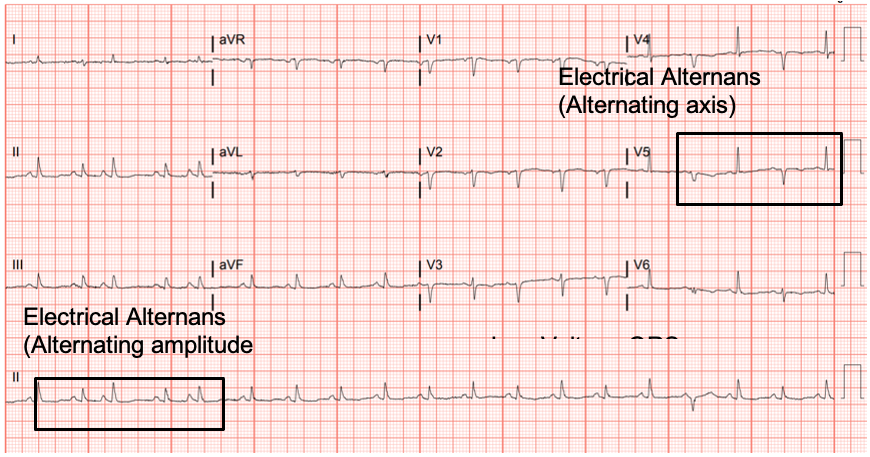

Supplement: Supplementary file 3 [file jetem-6-2-v8-supp3.png]

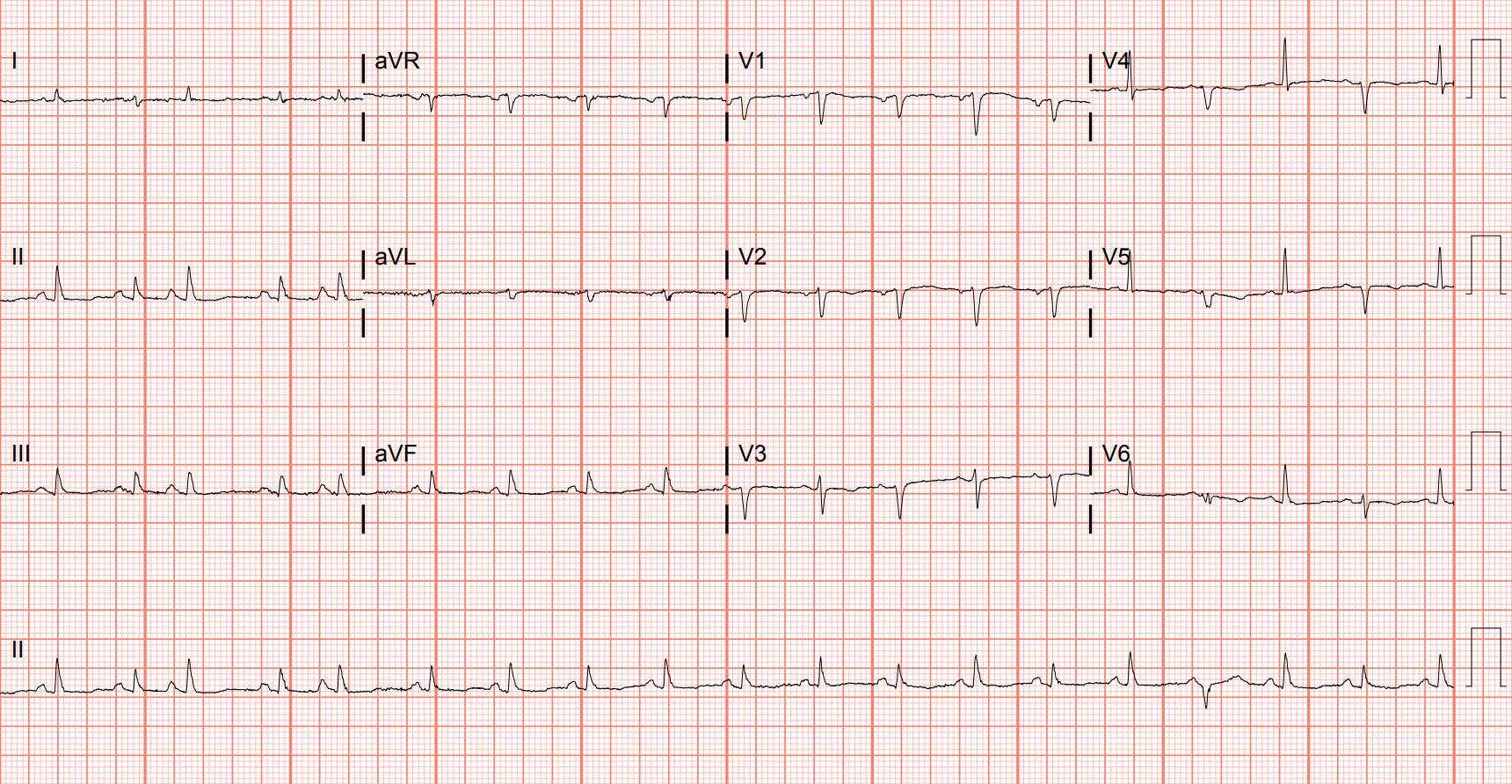

Supplement: Supplementary file 4 [file jetem-6-2-v8-supp4.jpg]

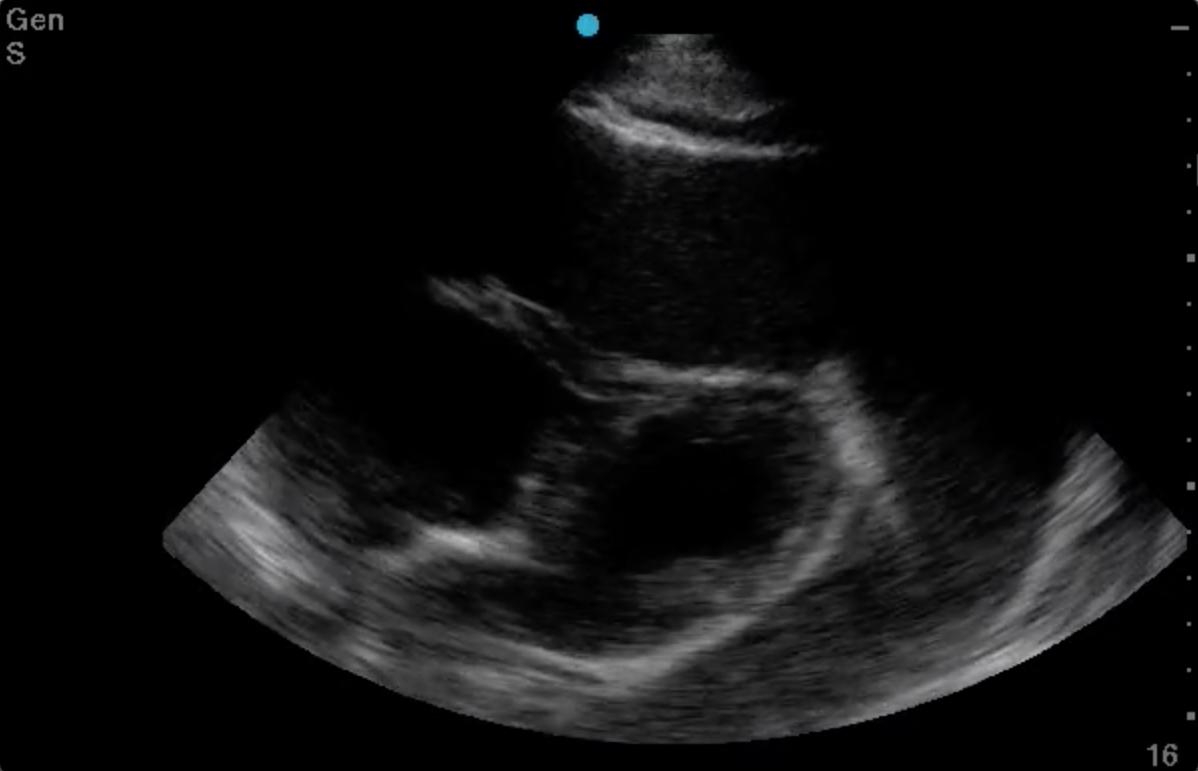

Supplement: Supplementary file 6 [file jetem-6-2-v8-supp6.png]
